# Oxidation of tricyclic antidepressant drugs with chloramine-T in acidic solutions: kinetic, mechanistic and thermodynamic studies

**DOI:** 10.1186/2193-1801-2-30

**Published:** 2013-01-30

**Authors:** Anu Sukhdev, Puttaswamy Puttaswamy

**Affiliations:** Department of Post-Graduate Studies in Chemistry, Bangalore University, Central College Campus, Bangalore, 560 001 India

**Keywords:** Tricyclic antidepressants, Chloramine-T, Oxidation-kinetics, Acid solutions

## Abstract

The kinetics of the oxidation of two tricyclic antidepressants (TCA) namely, imipramine (IMP) and clomipramine (CLM) with sodium N-chloro-p-toluenesulfonamide or chloramine-T (CAT) in HClO_4_ medium was studied at 300 K. The two reactions followed identical kinetics with a first-order dependence of rate on [CAT]_o_ and fractional order dependence on [TCA]_o_. The reaction is catalyzed by H^+^ ions with a fractional order dependence. The reaction was studied at different temperatures and activation parameters were evaluated. The reaction constants involved in the mechanism were computed. The solvent isotope effect was studied using D_2_O. Addition of p-toluenesulfonamide retards the reaction rate. The rate increased with decreasing dielectric constant of the medium. Variation of ionic strength of the medium and addition of halide ions (Cl^-^ or Br^-^) showed no effect on the rate. The stoichiometry of the reaction was found to be 1:1 and the oxidation products were identified as imipramine-5-N-oxide and clomipramine-5-N-oxide. The rate of oxidation of IMP is faster than CLM. The observed results have been explained in terms of a mechanism and a relevant rate law has been deduced.

## Background

Tricyclic antidepressants (TCA) are pharmaceutically important compounds and are widely used for the treatment of psychiatric patients suffering from clinical depression Goodman and Gilman (2001). The main function of these drugs is to block the reuptake of the neurotransmitters in the central nervous system (Morton 1991; British Pharmacopoeia 1988). Imipramine hydrochloride (IMP) and Clomipramine hydrochloride (CLM) are important compounds of this group of drugs. Inspite of the importance of these drugs, a review of literature reveals the absence of comprehensive studies on the solution behaviour of these drugs especially on their oxidation-kinetics and mechanisms. In biochemical reactions, kinetic knowledge is used to optimize the reaction conditions in order to unfold the mechanistic picture of the particular redox system which remains an area of interest and importance. Hence, such a study could throw some light on the mechanism of metabolic conversions of these drugs in the biological systems. It was, therefore, found to be of interest and important to investigate the mechanism of oxidation of IMP and CLM with halogen +1 oxidant kinetically.

Many reports are available in literature on the oxidative degradation of biological substrates by hypochlorous acid. Hence it was intended to study the oxidation kinetics of IMP and CLM drugs with HOCl as an oxidant, since it is a biologically more relevant oxidant. It is also reported that organic N-haloamines resemble hypohalites in its oxidative mechanistic behavior and although less familiar, they are more stable than hypohalites. Organic N-haloamines are finding applications as oxidants, disinfectants and antiseptics Bishop and Jennings (1958; Morris et al. 1948). In addition, both haloamines and hypohalites contain halogen in the +1 state.

The chemistry of organic N-haloamines is of interest due to their diverse behavior (Campbell and Johnson 1978). As a result, these reagents interact with a wide range of functional groups affecting an array of molecular transformations (Banerji et al. 1987; Armesto et al. 1998; Agnihotri 2005; Kolvari et al. 2007; Puttaswamy and Shubha 2009). The prominent member of this class of compounds is sodium N-chloro-p-toluenesulfonamide or chloramine-T (CAT). It is a mild, efficient, stable, non toxic and inexpensive oxidant. On the other hand, hypochlorous acid is not commercially available since it is highly unstable and has to be prepared and standardized every hour afresh. Because of these reasons, we have opted CAT as an oxidant. Hence, the present study gives an impetus as the substrates are potent drugs since the oxidative behavior of CAT is quite similar to hypochlorous acid. Therefore, we report herein the results of investigations on the kinetics and mechanism of oxidation of IMP and CLM in acid medium. The studies are also extended to deduce the appropriate rate law based on the kinetic results. The remarkable advantage in the course of this research is that the optimum conditions for the facile oxidation of TCA to 5-N-oxides were established.

## Results and discussion

Our preliminary experiments revealed that there is no reaction between substrate and perchloric acid under the experimental conditions employed. This ruled out the possibility of HClO_4_ as an oxidizing agent in the present case. Hence, it can be said that CAT is only involved in the oxidation of the drug. The kinetics of oxidation of imipramine and clomipramine with CAT was investigated at several initial concentrations of the reactants in HClO_4_ medium at 300 K. Under comparable experimental conditions, the similar oxidation kinetic behaviour was observed for both the drugs.

### Kinetic results

Under pseudo-first order conditions of [TCA]_o_ >> [CAT]_o_ at constant [TCA]_o,_ [HClO_4_] and temperature, plots of log [CAT] vs time were linear (*R*^*2*^ > 0.9927), indicating a first order dependence of the rate on [CAT]_o_. The values of pseudo-first order rate constants (*k*^*/*^ s^-1^) are given in Table 1. The values of *k*^*/*^ remain unaffected with a change in [CAT]_o,_ confirming the first order dependence on [CAT]_o_. The rate increased with increase in [TCA]_o_ (Table 1). Plots of log *k*^/^ vs log [TCA] were linear (*R*^*2*^ >0.9978) with slopes of 0.84 and 0.78 for IMP and CLM, respectively, indicating a fractional-order dependence of rate on [TCA]_o_. The fractional order with respect to the substrate, presumably results from a complex formation between oxidant and substrate prior to the formation of products. Indeed, in the present case, it is to be noted that the plots of 1 / *k*^*/*^ vs 1 / [TCA] were linear (*R*^*2*^ > 0.9994) having a y-intercept which is in agreement with such a complex formation. This establishes the fractional order dependence on [TCA]_o_. The reaction rates are enhanced with increase in [HClO_4_] (Table 1). Plots of log *k*^*/*^ vs log [HClO_4_] were linear (*R*^*2*^> 0.9959) with slopes equal to 0.38 and 0.47 for IMP and CLM respectively, showing a fractional-order dependence of rate on [HClO_4_].

**Table 1:** **Effect of varying CAT, TCA and HClO**_**4**_**concentrations on the rate of reaction at 300 K**

10^4^ [CAT]_o_	10^3^ [Substrate]_o_	10^3^ [HClO_4_]	10^4^***k***^***/***^ (s^-1^)	
(mol dm^-3^)	(mol dm^-3^)	(mol dm^-3^)	IMP	CLM
1.0	4.0	2.9	7.62	4.94
2.0	4.0	2.9	7.54	4.80
4.0	4.0	2.9	7.65	4.89
8.0	4.0	2.9	7.59	4.96
10.0	4.0	2.9	7.50	4.83
2.0	1.0	2.9	1.92	1.58
2.0	2.0	2.9	3.73	3.27
2.0	4.0	2.9	7.54	4.80
2.0	8.0	2.9	12.0	8.32
2.0	12.0	2.9	17.4	11.5
2.0	4.0	1.0	5.94	3.16
2.0	4.0	2.9	7.54	4.80
2.0	4.0	5.0	9.60	5.62
2.0	4.0	10.0	11.9	7.11
2.0	4.0	15.0	13.7	9.59

**Table 2:** **Effect of varying p-toluenesulfonamide (TsNH**_**2**_**or PTS) on the rate of reaction at 300 K**^**a**^

10^3^ [PTS]	10^4^***k***^***/***^ (s^-1^)	
(mol dm^-3^)	[IMP]	[CLM]
2.0	7.24	4.03
4.0	5.24	3.42
5.0	4.79	2.86
8.0	3.98	1.94

^a^[CAT]_o_ = 2.0 x 10^-4^ mol dm^-3^; [Substrate]_o_= 4.0 x 10^-3^ mol dm^-3^;[HClO_4_] = 2.9 x 10^-3^ mol dm^-3^.

**Figure 1:**
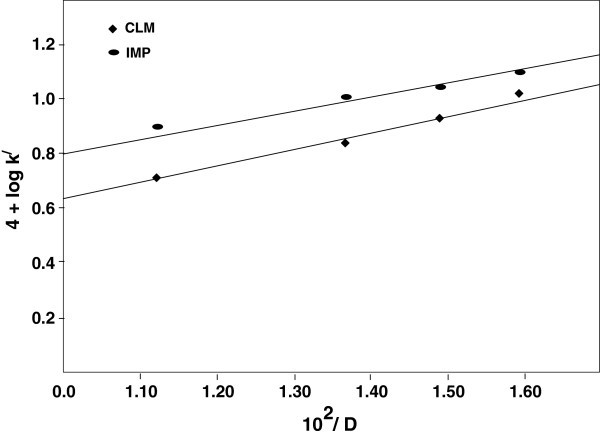
**Plots of log*k***^***/***^**versus 1 / D.**

Addition of p-toluenesulfonamide (PTS or TsNH_2_; 2.0 x 10^-3^ – 8.0 x 10^-3^ mol dm^-3^), the reduction product of CAT, retards the rate of the reaction in both cases (Table 2). Further, log-log plots of *k*^*/*^ vs [PTS] were linear (R^*2*^> 0.9994) with negatives slopes of 0.36 and 0.31 for IMP and CLM respectively, indicating a negative fractional-order dependence of the rate on [PTS]. It also indicates that PTS is involved in a fast pre- equilibrium to the rate-determining step (rds) in the proposed reaction scheme. In order to find out the nature of the reactive species, the dielectric constant (D) of the medium was varied by adding MeOH (0 - 30% v/v) to the reaction mixture by keeping all other experimental conditions constant. An increase in the rate was noticed with increase in MeOH content in both cases. Plots of log *k*^/^ vs 1/ D were linear (R^2^ > 0.9920) with positive slopes. The results are graphically represented in Figure 1. The values of dielectric constant of MeOH-H_2_O mixtures of different compositions are available in the literature Akerloff (1932) Controlled experiments with MeOH indicated that its oxidation by CAT was negligible (< 2 %) under the present set of experimental conditions. However, the rate constants were corrected to present only the oxidation of IMP and CLM.

Effect of ionic strength of the reaction system on the rate of the reaction was studied by adding 0.3 mol dm^-3^ NaClO_4_ solution to the reaction mixture. It was noticed that there was no remarkable change on the rate of the reaction. Hence, no attempt was made to keep the ionic strength of the system constant for kinetic runs. The rate remained constant with the addition of Cl^-^ or Br- ions in the form of NaCl or NaBr (2.0 x 10^-2^ mol dm^-3^) had no significant effect on the rate of reaction, indicating that no inter halogen compound or free chlorine was formed. As a dependence of the rate on hydrogen ion concentration was noticed, solvent isotope studies were made. using D_2_O for both the drugs. Studies of the reaction rate in D_2_O medium for IMP and CLM revealed that *k*^/^ (H_2_O) was equal to 7.54 x 10^-4^ s^-1^ and 4.80 x 10^-4^ s^-1^, and *k*^/^ (D_2_O) was 9.20 x 10^-4^ s^-1^ and 6.38 x 10^-4^ s^-1^ respectively. The solvent isotope effect *k*^/^ (H_2_O) / *k*^/^ (D_2_O) was found to be 0.82 and 0.75 for IMP and CLM. The reaction rates were determined at different temperatures (290–310 K), keeping the other experimental conditions the same. Based on the Arrhenius plots of log k^/^ vs 1/T (R^2^ > 0.9908), activation energy and other activation parameters were computed for the overall reaction. All these results are summarized in Table 3. The oxidation reaction fails to induce the polymerization of the added acrylonitrile, indicating the absence of the formation of any free radical during the reaction sequence

**Table 3:** **Effect of varying temperature and values of thermodynamic parameters for the oxidation of IMP and CLM by CAT in acid medium**

Temperature	10^4^ k^/^ (s^-1^) (10^3^***k***_***3***_(s^-1^))^a^	
(K)	IMP	CLM
290	3.90 (1.66)	2.58 (1.11)
295	4.86 ( ---)	3.36 ( ---)
300	7.54 (2.50)	4.80 (1.43)
305	9.98 (3.30)	5.92(2.0)
310	15.8 (4.0)	9.40 (2.64)
*E*_*a*_ (kJ mol ^-1^)	38.3 (32.6)	46.7 (38.5)
*∆H*^*≠*^ (kJ mol^-1^)	35.8 (30.1)±0.01	44.2 (35.8)±0.01
*∆G*^*≠*^ (kJ mol^-1^)	91.5 (88.6)±0.09	92.7 (90.0)±0.002
*∆S*^*≠*^ (JK^-1^ mol^-1^)	-185 (-194)±0.03	-161 (-180)±0.08
*Log A*	6.94±0.06	8.56±0.24

^a^Values in the parantheses refer to the rate-determining step.

[CAT]_o_ = 2.0 x 10^-4^ mol dm^-3^; [TCA]_o_ = 4. 0 x 10^-3^ mol dm^-3^; [HClO_4_] = 2.9 x 10^-3^ mol dm^-3^.

### Reactive species of chloramine-T

Chloramine-T (TsNClNa) acts as a mild oxidant in both acidic and alkaline media Campbell and Johnson (1978). In general, CAT undergoes a two electron change in its reactions forming the reduction products, PTS and sodium chloride Bishop and Jennings (1958). The oxidation potential of CAT-PTS redox couple is pH dependant Campbell and Johnson (1978; Murthy and Rao 1952) and it decreases with increase in pH of the medium (the redox potential E_redox_ of CAT-PTS couple is 1.138, 0.778, 0.614 and 0.50 V at pH 0.65, 7.0, 9.7 and 12, respectively). Chloramine-T behaves as a strong electrolyte in aqueous solutions [Hardy and Johnston 1973 and depending on the pH of the medium, it furnishes Bishop and Jennings (1958; Morris et al. 1948,1973; Pryde and Soper 1931) different reactive species in solutions:

Therefore, the possible oxidizing species in acidified CAT solutions are TsNHCl, TsNCl_2_, HOCl and possibly H_2_O^+^Cl. Also, Narayan and Rao (1983) and Subhashini *et* al. (1985) have reported that chloramine-T can be protonated further at pH 2 . If dichloramine-T (TsNCl_2_) were to be the reactive species, then the rate law predicts a second-order dependence of rate on [CAT]_o_ which is contrary to the experimental observations. In the present study, the rate of the reaction increases with increase in [H^+^] but it is retarded by the added p-toluenesulfonamide (TsNH_2_). Further, chloramine-T contains a polar N-Cl bond as the source of positive chlorine Cl^+^ species, which forms the conjugate acid TsNHCl, in acidic solutions. This conjugate acid with N-Cl bond intact interacts with H_3_O^+^ to form the reactive oxidant species, H_2_O^+^Cl, and the p-toluenesulfonamide (TsNH_2_).

### Reactive species of TCA

**Scheme 1:**
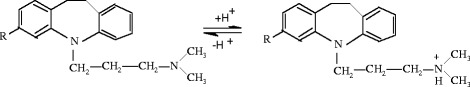
**Reactive Species of TCA.**

The pKa of substrates IMP and CLM were reported as 9.5 and hence these drugs are basic in nature. Consequently, they get readily protonated in acidic pH. The present redox system was studied around pH 3, which is less than pKa of the drugs. Hence these drugs get protonated Newton and Kluza (1978) is shown in Scheme 1:

In the present case, form B of the substrate is considered as the substrate reactive species.

### Reaction scheme

**Scheme 2:**
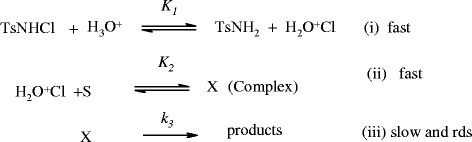
**A general scheme for the oxidation of TCA with CAT in acid medium.**

Based on the preceding discussion and experimental observations, a tentative mechanism (Scheme 2) for the oxidation of IMP and CLM with CAT in acid medium has been proposed.

 shown in Scheme 2.

**Scheme 3:**
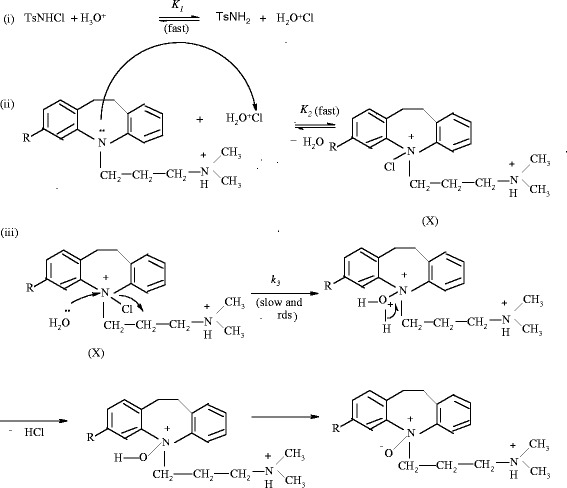
**A detailed mechanistic interpretation for the oxidation of TCA-CAT redox system in acid medium.**

In Scheme 3, S is the substrate and X is the intermediate complex species whose possible structure in shown in Scheme 3 in which a detailed mechanistic interpretation of TCA – CAT reaction in acid medium is presented. In a fast pre equilibrium step, the protonation and subsequent hydrolysis of TsNHCl yields the reactive oxidizing species H_2_O^+^Cl, with the elimination of TsNH_2_. In the next fast equilibrium step, lone pair of electrons on the nitrogen atom of the substrate reacts with positive chlorine of the oxidizing species to form an intermediate complex (X). Further, in the next slow and rate-determining step, X undergoes a nucleophilic attack of water molecule to yield the final products 5-N ➝ oxides with the elimination of a molecule of HCl.

### Kinetic rate law

If [CAT]_t_ represents the total concentration of [CAT], then1

From steps (i) and (ii) of Scheme 2,23

By substituting for [TsNHCl] and [H_2_O^+^Cl] from eq 2 and eq 3 respectively into eq 1 and solving for X, we get4

From the slow and rate-determining step (step(iii) of Figure 1.5

By substituting for [X], from eq 4 into eq 5, the following rate law is obtained:6

Rate law (6) is in good agreement with the experimental results, wherein a first-order dependence of rate on [CAT]_o_, fractional-order dependence each on [TCA]_o_ and [H^+^], and an inverse-fractional order on [TsNH_2_] was observed.

Since Rate = *k*^*/*^ [CAT]_t_, under pseudo-first order conditions of [CAT]o <<< [TCA]_o_, eq 6 can be transformed as eq 7, eq 8 and eq 9.789

According to eq 8 and eq 9, in order to deduce equilibrium and decomposition constants, the reaction has been studied in presence of 2.0 x 10^-3^ mol dm^-3^ p-toluenesulfonamide (TsNH_2_) by varying the concentrations of TCA and HClO_4_ in the range given in Table 1.

**Figure 2:**
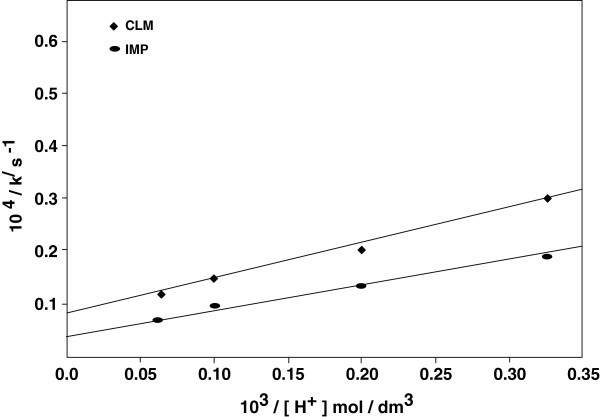
**Double reciprocal plots of 1 /*k***^***/***^**versus 1/ [H**^**+**^**] at constant [TsNH**_**2**_**] = 2.0 x10**^**-3**^**mol dm**^**-3**^**.** Other experimental conditions are as in Table 1.

From eq 8, plots of 1 / *k*^*/*^ vs 1 / [H^*+*^] at constant [TCA] and [TsNH_2_] were linear (Figure 2; *R*^*2*^ > 0.9907) with

**Figure 3:**
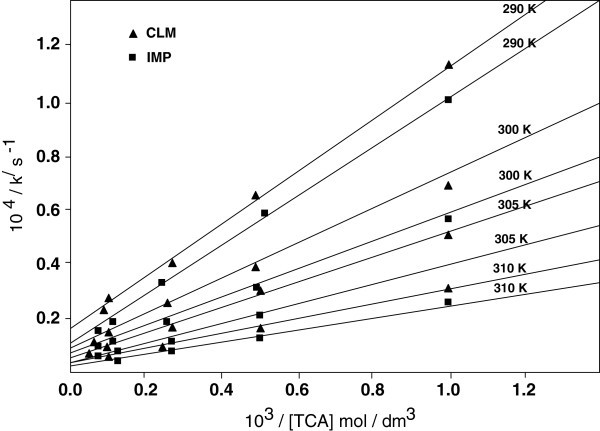
**Double reciprocal plots of 1 /*k***^***/***^**versus 1 / [TCA] at constant [TsNH**_**2**_**] = 2.0x10**^**-3**^**mol dm**^**-3**^**.** Other experimental conditions are as in Table 1.

From eq 9 plots of 1/k^/^ versus 1/ [TCA] at constant [H^+^] and [TsNH_2_] were linear (Figure 3; *R*^*2*^*=* 0.9910) with

From the slopes and the intercepts of the above plots, the values of equilibrium constants *K*_*1*_ and *K*_*2*_, and the decomposition constant k_3_ were found to be 0.6 and 0.5, 84 and 151 mol dm^-3^ and 5.0 x 10^-3^ and 1.66 x 10^-3^ s^-1^ for IMP and CLM, respectively. The proposed scheme and the derived rate law are also supported by the experimental observations discussed below.

### Michaelis-menten kinetics

Since the rate was fractional-order in [TCA]_o_, Michaelis-Menten kinetics House (2007) were adopted to study the effect of substrate on the rate at different temperatures. The decomposition constant *k*_*3*_ values were determined by varying the concentration of both the drugs in the range given in Table 1 at different temperatures (290 – 310 K) at constant [TsNH_2_] = 2.0 x 10^-3^ moldm^-3^. The activation parameters for the rate-determining step were evaluated using Arrhenius plots of log *k*_*3*_ vs 1 / T (*R*^*2*^ > 0.9959). All these results are compiled in Table 3. The proposed mechanism and the derived rate law are supported by the following experimental findings:

### Solvent isotope effect

As expected for a H^+^ catalyzed reaction, the rate of the reaction increased in D_2_O medium and hence the proposed mechanism is supported by this observation. For a reaction involving a fast equilibrium H^+^ or OH^-^ transfer, the rate increases in D_2_O since D_3_O^+^ and OD^-^ which are stronger acid and stronger base (~ 2–3 times greater), respectively, than H_3_O^+^ and OH^-^ ions Collins and Bowman (1970; Kohen and Limbach 2006). The increase of reaction rate with D_2_O observed in the present studies and the solvent isotope effect which is *k*^/^ (H_2_O) / *k*^/^ (D_2_O) < 1 conform to the above theory. However, the magnitude of acceleration in D_2_O is small compared to the expected value, which can be attributed to the fractional order dependence on [H^+^].

### Dielectric constant effect

A change in the solvent composition by varying the methanol content in methanol–water affects the reaction rate. The effect of solvent on the reaction kinetics has been described in detail in the well-known publications of Moelwyn- Hughes (1947), Benson (1960), Frost and Pearson (1961), Laidler and Eyrings (1940), Amis (1966), and Entelis and Tiger (1976). For the limiting case of zero angle of approach between two dipoles or an ion dipole system, Amis (1966) has shown that a plot of log *k*^*/*^ versus 1 / D gives a straight line, with a negative slope for a reaction between a negative ion and a dipole or between the dipoles, while a positive slope results for a positive ion-dipole interaction. In the present observations, plots of log *k*^*/*^ vs 1 /D were linear with positive slopes and hence the later concept agrees where a positive ion and a dipole are involved in the rate-determining step of the proposed scheme (Scheme 3).

### Ionic strength effect

The proposed reaction mechanism is also evinced by the observed effect of ionic strength on the rate of the reaction. The primary salt effect on the reaction rates has been described by Bronsted and Bjerrum Laidler (1965) theory. According to this concept, the effect of ionic strength (μ) on the rate of a reaction involving two ions is given by the relationship10

where A and B are the reacting ions, Z_A_ and Z_B_ are the charges on the respective species, *k*^/^ and *k*_*o*_ are the rate constants in the presence and in the absence of the added electrolyte, respectively. Equation 10 shows that a plot of log *k*^*/*^ versus μ^1/2^ would be linear yielding a slope 1.02 Z_A_Z_B_ and an intercept log *k*_*o*_. As the slope of the line depends on Z_A_Z_B,_ ie, charges of the reacting ions, three special cases may arise: (i) if A and B have the same charges, Z_A_ Z_B_ will be positive and the rate constant *k*^*/*^ increases with √μ; (ii) if A and B have opposite signs, Z_A_ Z_B_ will be negative and the rate constant *k*^*/*^ decreases with √μ; and (iii) if either A or B is uncharged, Z_A_ Z_B_ is equal to zero and *k*^*/*^ is independent of the ionic strength of the solution. In the present case, a positive charge and a neutral molecule is involved in the rate-determining step (step (iii) of Scheme 3). Hence, variation of the ionic strength of the medium does not alter the rate in both the cases clearly conform to the above theory (case (iii)).

### Relative reactivity of drugs

The relative reaction rates and activation energies indicate that the IMP oxidation is faster when compared to CLM. Since chlorine is present at the meta position, the electron-withdrawing inductive effect dominates and thus it deactivates the ring which makes the nitrogen atom of the ring less reactive towards the reactive oxidant species. Hence, the rate of oxidation of CLM is comparatively slower than IMP.

### Activation parameters

The proposed mechanism and the related rate law are supported by the moderate values of energy of activation and other activation parameters. The fairly high positive values of ∆G^≠^ and ∆H^≠^ indicate that the transition state is highly solvated, while the high negative ∆S^≠^ suggests the formation of a rigid associative transition state with a reduction in the degrees of freedom of molecules in both the drugs. The values of *∆G*^*≠*^ are almost the same in both the cases suggesting that the oxidation of IMP and CLM with CAT proceeds by a similar mechanism.

## Conclusions

Based on the present research work, the following conclusive remarks are drawn:

The oxidation reaction follows similar kinetic patterns for both the drugs.

The reaction obeys the experimental rate law: rate  =  k^/^ [CAT]_o_  [HClO_4_]^y^ [PTS]^- z^, where x,y,z <1.

The rate of oxidation of IMP is faster than CLM.

The thermodynamic parameters and reaction constants were evaluated.

Reaction mechanism (Scheme) and the rigorous kinetic modeling proposed, accord results fitting well with the experimental data.

In the course of this research, optimum conditions for the facile oxidative conversion of IMP and CLM to the corresponding 5-N oxides were established.

## Methods

### Materials

Chloramine-T (Merck) was purified by the method of Morris et al. (1948). An aqueous solution of CAT was prepared, standardized iodometrically and stored in amber colored stoppered bottles to prevent any of its photochemical deterioration. The concentration of stock solutions was periodically determined. The drugs imipramine hydrochloride and clomipramine hydrochloride of analytical grade gifted by R.L. Fine chemicals pvt. Ltd, Bangalore, India, was used as received. Aqueous solutions of desired strength of these drugs were prepared before use. Heavy water (D_2_O 99.4%) was supplied by Babha atomic research centre, Mumbai, India. All chemicals and reagents used were of Anala R grade and doubly distilled water was used throughout the work.

### Kinetic procedure

The kinetic runs were performed under pseudo-first order conditions with a large excess of substrate over oxidant in acid medium. Unless specificied otherwise, all the kinetic runs were carried out at 300 K. Detailed kinetic procedure followed is similar to that reported earlier (Mendham et al. 2000). The progress of the reaction was monitored by iodometric determination of unreacted CAT in measured aliquots (5 ml each) of the reaction mixture at different intervals of time. The course of the reaction was studied for more than two half-lives. The pseudo-first order rate constants (k^/^ s^-1^), were computed using the graphical methods by plotting log [CAT] vs time. Duplicate kinetic runs showed that the rate constants were reproducible with an accuracy of ± 6% error. All regression co-efficients (R^2^) calculations were performed with fx-100W scientific calculator.

**Scheme 4:**
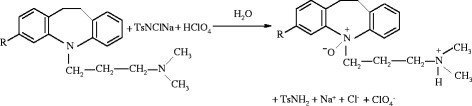
**Stoichiometric equation**. Here R = -Cl for CLM and R= -H for IMP, and Ts= -CH_3_C_6_H_4_SO_2_-.

Reaction mixtures containing varying proportions of CAT and substrates were equilibrated at 300 K in presence of 2.9 x 10^-3^ mol dm^-3^ HClO_4_ for 24h. An iodometric determination of the residual oxidant showed that one mole of CAT consumed per mole of the substrate, confirming the following stoichiometry (Scheme 4):

### Product analysis

**Figure 4:**
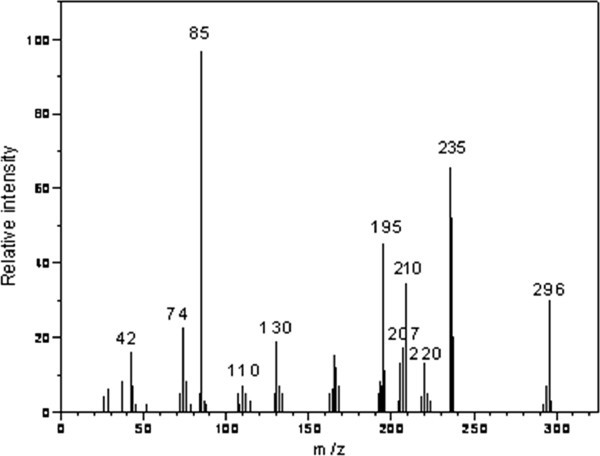
**GC – MS of imipramine-5N-oxide with its molecular ion peak at 296 amu.**

**Figure 5:**
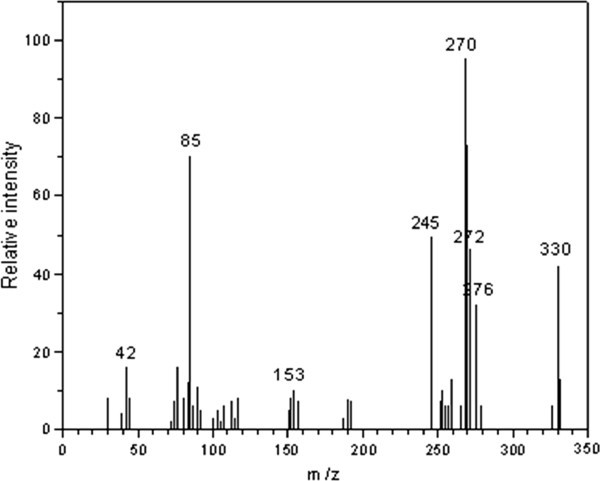
**GC – MS of clomipramine-5N-oxide with its parent molecular ion peak at 330 amu.**

The reaction mixtures (1 mole of substrate and 1 mole of CAT in the presence of 2.9 x 10^-3^ mol dm-^3^ HClO_4_) were allowed to progress for 6 – 8 hours under stirred condition at 300 K. After completion of the reaction (monitored by TLC), the reaction products were neutralized with NaOH. The oxidation products of TCA were subjected to spot tests and chromatographic analysis (TLC technique), which revealed the formation of imipramine-5-N-oxide and clomipramine-5-N-oxide as the oxidation products of imipramine hydrochloride and clomipramine hydrochloride, respectively. These oxidation products were separated by column chromatography on silica gel (60–120 mesh) (chloroform:methanol) as the solvent system. The products were confirmed by GC-MS analysis. GC-MS data were obtained on a 17A Shimadzu gas chromatograph with a QP-5050A Shimadzu mass spectrometer. The mass spectrum was obtained using the electron impact ionization technique. The mass spectra showed a molecular ion peak at 296 and 330 amu, clearly confirming imipramine-5-N-oxide and clomipramine-5-N-oxide, respectively (Figures 4 and 5). All other peaks observed in GC-MS can be interpreted in accordance with the observed structure. The formation of N-O bond in both the cases was confirmed by IR spectra: IR was recorded on a Shimadzu FT-IR-8400 spectrophotometer with KBr pellets. It showed an absorption peak at 1255 cm^-1^ and 1262 cm^-1^ for imipramine-5-N-oxide and clomipramine-5-N-oxide, respectively, which is due to N=O stretching (expected range is 1250 ± 50cm^-1^) in both the products. The reduction product of CAT, p-toluenesulfonamide (PTS or TsNH_2_), was extracted with ethyl acetate and detected by paper chromatography Jagadeesh RV (2008). Benzyl alcohol saturated with water was used as the solvent with 0.5% vanillin in 1% HCl solution (in ethanol) as spray reagent (R_f_ = 0.905). It was also observed that there was no further oxidation of these products under the present kinetic conditions. Consequently, the present redox system developed was found to be an efficient method and the involvement of cost effective reagents makes the reaction simple and expedient for scaling this method for the industrial operation to synthesize imipramine-5-N-oxide and clomipramine-5-N-oxide with suitable modifications. Hence, this protocol for the synthesis of imipramine-5-oxide and clomipramine-5-oxide will be a valuable addition to the existing methods.
